# A Convolutional Code-Based Sequence Analysis Model and Its Application

**DOI:** 10.3390/ijms14048393

**Published:** 2013-04-16

**Authors:** Xiao Liu, Xiaoli Geng

**Affiliations:** College of Communication Engineering, Chongqing University, 174 ShaPingBa District, Chongqing 400044, China; E-Mail: xiaoliweiliang408@163.com

**Keywords:** convolutional code, degeneracy, codon, informational unit, code distance, characteristic average code distance, GC content, taxonomy

## Abstract

A new approach for encoding DNA sequences as input for DNA sequence analysis is proposed using the error correction coding theory of communication engineering. The encoder was designed as a convolutional code model whose generator matrix is designed based on the degeneracy of codons, with a codon treated in the model as an informational unit. The utility of the proposed model was demonstrated through the analysis of twelve prokaryote and nine eukaryote DNA sequences having different GC contents. Distinct differences in code distances were observed near the initiation and termination sites in the open reading frame, which provided a well-regulated characterization of the DNA sequences. Clearly distinguished period-3 features appeared in the coding regions, and the characteristic average code distances of the analyzed sequences were approximately proportional to their GC contents, particularly in the selected prokaryotic organisms, presenting the potential utility as an added taxonomic characteristic for use in studying the relationships of living organisms.

## 1. Introduction

Biological science appears to be independent of communication engineering in the traditional sense, but both systems involve information transmission that requires efficiency and anti-jamming capability. As both systems have error-correction mechanisms, similarities between biological mechanisms and modern communication theory, especially in the area of error correction, has attracted the interest of many scholars to the study of the combined fields [1–9].

Until now, some models based on communication theory have been established to parallel DNA processes, such as a model based on the error control coding theory and the central dogma of genetics [4], a model for gene expression based on the assumption that the ribosome decodes mRNA sequences using the 3′-end of the 16S rRNA molecule as a one-dimensional codebook [10] and a mathematical model of genetic information storage and transmission between proteins [11]. Further research on living systems has also been emphasized, such as the concept of robustness of living systems [12] and the similarity of DNA sequences [13]. Research results have been used to improve the work in related fields, such as in the error control coding theory applied in microarray data analysis [14], biology and biomolecular computing [15], biodetection and classification [16], multiclass classification in cancer diagnosis [17] and transcription factor classification [18]. These results illustrate the significance and need for the study of biological problems in terms of the error control coding theory.

May *et al.*[4] applied a block code model to the analysis of mRNA translation initiation, such that the last 13 bases of the 16S rRNA of *Escherichia coli K-12* were used as a template to generate parity bits and then a set of code words obtained to decode the genetic sequence. A genetic algorithm-based method was used with considerable success in constructing convolutional code models for ribosomal binding site recognition [3]. Following their study, Ponnala *et al.*[19] applied analytical methods to identify good generators for a convolutional code model for studying translation initiation in *Escherichia coli K-12*; 16S rRNA was also used for designing a generator. Nevertheless, there are several remaining questions worthy of further discussion:

(i) The work of Ponnala *et al.*[19] produced a better result using a block code model, but a convolutional code model is another model that provides better performance in many cases in a coding system of communication engineering. This observation indicates that a convolutional code model approach should be studied more extensively.
(ii) Researchers have discovered the effect of codon context on the expression and efficiency of the translation of some codons [20,21], but the effect of the adjacent nucleotides is not considered sufficiently in these models. Thus, a convolutional code model, which contains the effect of adjacent symbols, should be more suitable for studying DNA encoding than a block code model, which only considers the effect of the present symbols.
(iii) A nucleotide in a DNA sequence is usually treated as an independent informational unit in the traditional methods, but codon functions in the process of translation imply that a codon itself could be treated as an informational unit [22].
(iv) In addition, the degeneracy of the codons is quite interesting, as the existence of degeneracy provides more stability in genetic processes, such that a gene mutation of one nucleotide may result in another codon of the same amino acid. Thus, this feature of codon should be an important feature or consideration in designing an analytical model.

In this article, a convolutional code-based model for DNA sequences is proposed, in which a codon is treated as an informational unit and the generator matrix is designed based on codon degeneracy. Without consideration of a specific segment of a genetic sequence, such as 16S rRNA, the proposed model is species-independent, as it addresses a universal biological feature.

## 2. Results and Discussion

**Figure 1:**
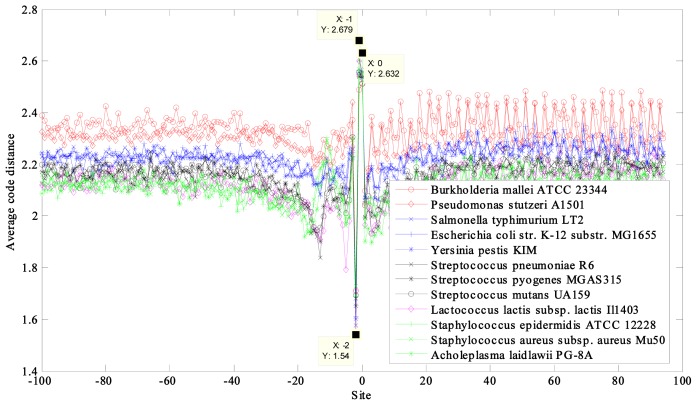
Curves of average code distance of the 12 prokaryotes near initiation site.

**Figure 2:**
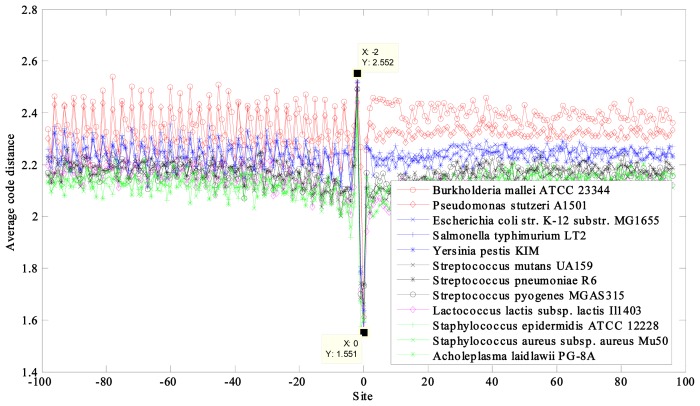
Curves of average code distance of the 12 prokaryotes near termination site.

**Figure 3:**
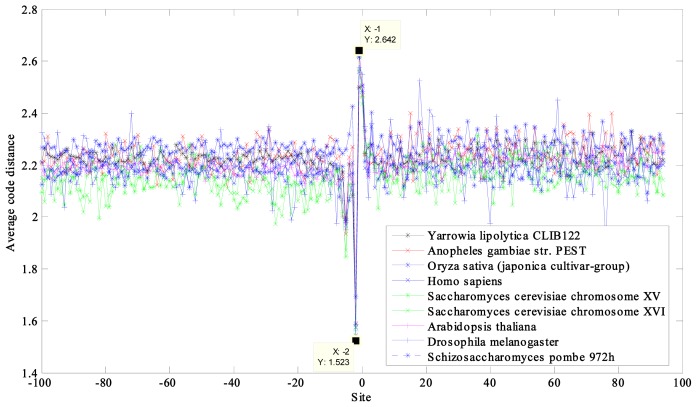
Curves of average code distance of the nine eukaryotes near initiation site.

**Figure 4:**
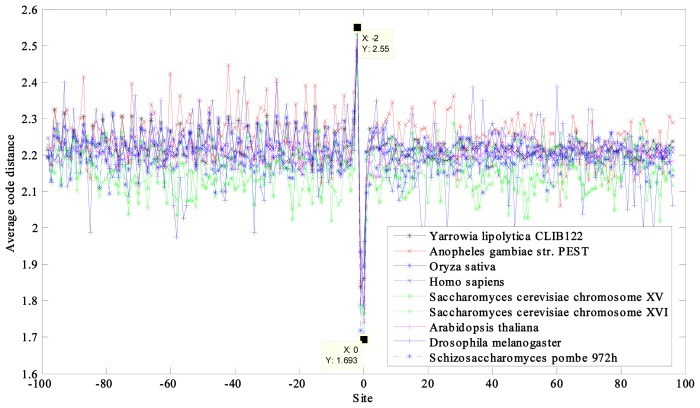
Curves of average code distance of the nine eukaryotes near termination site.

The average code distances of 12 prokaryotic and nine eukaryotic DNA sequences near the initiation and termination site were calculated and plotted (Figures 1, 2, 3, 4). Their characteristic average code distances (CACD; see analysis Step 5 in Section 3.2) were calculated, as were CACDs based on May *et al.*’s (5, 2) block code and both sets of results listed for comparison in Tables 1 and 2, respectively (see analysis Step 3 in Section 3.2 for a basic definition of code distance.)

### 2.1. Region near the Initiation Site

For the prokaryotic DNA sequences, a significant trough appeared at site −2 and crests appeared near the initiation site at site −1 and 0 in every curve (Figure 1). Furthermore, a clear upward heave appeared near site −11 for most of the prokaryotic DNA sequences, and curves with lower average code distance displayed stronger changes. However, two sequences, from *Pseudomonas stutzeri A1501* and *Burkholderia mallei ATCC 23344*, which showed higher average code distance than the other sequences, displayed slight downward changes (Figure 1, two red curves). The fluctuation near site −11 was attributed to the existence of a Shine-Dalgarno (SD) sequence, whose location was in the range of 5–13 nucleotides before the initiation site and with a relatively high purine (G and A) content [23]. This feature was essentially similar to the use of a preamble in a communication system.

Similar results were observed in the results from eukaryotic DNA sequences, with significant troughs appearing at site −2 and crests at site −1 in each curve (Figure 3). However, changes near the site −11, as observed in the analyzed prokaryotic DNA sequences, were not present. This was attributed to the absence of SD sequences in eukaryotes.

Finally, these results exhibited the efficiency of this method for identifying translation initiation sites and the location of SD sequences.

### 2.2. Region near the Termination Site

For the prokaryotic DNA sequences, significant troughs appeared at site −2 and crests appeared at site 0 in each curve near the termination site (Figure 2). The location of the change was closely associated with the location of the stop codon.

This was similar to the results from the eukaryotic DNA sequences, whose curves also included remarkable troughs at the site −2 and crests at site 0 (Figure 4).

### 2.3. Period-3 Feature in Coding Region

A period-3 feature is remarkable in the coding regions of all of the sequences, especially the prokaryotic DNA sequences with higher average code distance. Some curves of the eukaryotic DNA sequences showed weak periodicity after the start codon and more obviously before the stop codon, such as in *Saccharomyces cerevisiae* (Figures 3 and 4).

This proposed model provided a clearer result in detecting periodicity in the coding regions than did the existing models.

### 2.4. Separating the Derived Curves into Groups

The curves derived from the prokaryotic and eukaryotic DNA were separated into groups, with the prokaryotic DNA sequences distinguished by differences in GC content. As CACD values were approximately proportional to the corresponding GC contents (see Table 1) and GC content is used as a basic feature for microorganism taxonomy (*i.e.*, similar GC content indicates a higher possibility of being close relatives) [24,25], it was attempted here to link CACD to taxonomy.

Positive examples here of GC content and CACD values indicating relatedness were *Pseudomonas stutzeri A1501* and *Burkholderia mallei ATCC 23344*. Their corresponding curves group together with GC contents of 63% and 68% and CACDs of 2.3031 and 2.3179, respectively. Their full lineage listed in the National Center for Biotechnology Information (NCBI) are “*cellular organisms, Bacteria, Proteobacteria, Gammaproteobacteria, Pseudomonadales, Pseudomonadaceae, Pseudomonas, Pseudomonas stutzeri group, Pseudomonas stutzeri*” and “*cellular organisms, Bacteria, Proteobacteria, Betaproteobacteria, Burkholderiales, Burkholderiaceae, Burkholderia, Burkholderia mallei*”, respectively. Yabuuchi *et al.*[26] transferred seven species, including *Burkholderia mallei*, to a new genus, *Pseudomonas*, based on 16S rRNA sequences, DNA–DNA homology values, cellular lipid and fatty acid composition and phenotypic characteristics. A synonym of *Burkholderia mallei* is defined as *Pseudomonas mallei* in the UniProt website. This pairing of species suggested that this new method could provide clues in identifying misclassified organisms and for grouping them together using these coding calculations.

Negative examples were also found, such that the green curves representing *Staphylococcus epidermidis ATCC 12228* and *Staphylococcus aureus* subsp. *aureus Mu50* (GC content of 32% in both) overlapped the curve representing *Acholeplasma laidlawii PG-8A* (GC content of 31%) because of their similar GC contents (Figure 1). Their CACDs were also very close, but the division of the first two species is *Firmicutes* and the third one *Tenericutes*, indicating that they are clearly different species.

For the eukaryotic DNA sequences, the derived curves separated into groups of various distances, but the relationship between CACD and GC content was a little irregular (Figure 3). One reason for this may have been that it was insufficient to attempt to reflect the complexity of eukaryotes simply in terms of the narrow range of their GC content (~30%–50%).

The positive examples of detecting relatedness by GC content and CACD values were *Saccharomyces cerevisiae* and *Schizosaccharomyces pombe*. Although the two yeasts are contained in *Ascomycota*, *Saccharomyces cerevisiae* is a primitive eukaryote. The CACD of *Schizosaccharomyces pombe* (GC content of 36%) was clearly higher than that of *Saccharomyces cerevisiae* (GC content of 38%, Table 2) and closer to that of the advanced eukaryotes, according to the present method. Both yeasts have been shown to be distant relatives, differing from each other in systematic classification, cell cycle, rRNA biosynthesis, gene structure and gene expression regulation. Furthermore, *Schizosaccharomyces pombe* is more similar to advanced eukaryotes in some aspects than the other yeast [27].

Here, the obtained results based on CACD values were better than those based on May *et al.*’s model [4], *i.e.*, our model provided a more linear relationship for prokaryotic DNA sequences and larger CACD distances. This difference in the results between these two models may have been caused by the fact that the other model was designed on the 16S rRNA of *Escherichia coli K-12* and was, thus, species-dependent.

## 3. Experimental Section

On receiving an input of *k* bits, a convolutional encoder produces an output of *n* (*n* > *k*) bits, which associates with not only the present *k* bits input, but also the previous *L*-1 group(s) of *k* bits input, where *L* is called the constraint length of this convolutional code [28]. A convolutional encoder contains a memory array, where each memory cell provides one output to be linearly combined. In fact, the coefficients of the linear combination determine whether a certain output is used. In a binary system, a coefficient of 1 means the output of one memory cell is used in the linear combination and 0 means it is not. In practice, the coefficients are brought together into a generator matrix.

### 3.1. Designing the (6,3,2) Convolutional Code Model

Luo *et al.*[29] showed the existence of the strong short-range correlation of adjacent bases. Cohen *et al.*[30] found that adjacent genes, in any orientation, are more likely to be co-expressed than non-adjacent genes. Kruglyak and Tang [31] showed that the expression patterns of adjacent genes are more often highly correlated than the expression patterns of randomly selected gene pairs. Marin *et al.*[32] found that short-range correlation phenomena in the yeast genome are related to the transcriptional orientation of nearest neighbor open reading frames (ORFs). Taken together, these studies stressed the effect of an informational unit from the nearest informational unit. Furthermore, according to the effects of codon context, it was supposed that a codon carried not only the genetic information of itself, but also a part of the genetic and error correcting information of its adjacent codons.

From these considerations, two new viewpoints were developed:

(i) A codon is treated as an informational unit, as a codon in the coding region is translated into an amino acid, which is different from using a single nucleotide. Thus, 3 or multiples of 3 should be used as the basic code length, and based on the short-range dominance of bases correlation [29], 2 was selected as the universal constraint length (*i.e*., *L* = 2), and the length of convolutional code was defined as 6. In other words, the encoder output depends on two contiguous codons, and the selected code is a (6,3,2) convolutional code. Every 3 nucleotides were used as a group of input and 6 symbols generated as outputs, which were simultaneously affected by both the present and previous inputs.
(ii) The design of the coefficients in the generator matrix was based on codon degeneracy, *i.e.*, the translated amino acid may be the same even for different codons (this is largely, but not entirely, confined to the third position of a codon, known as the wobble position). The wobble feature of synonymous codons reduces the influence of mutation on living systems, as a gene mutation of one nucleotide may result in another codon of the same amino acid. This phenomenon was considered an important feature in the design. We supposed that the first two codon nucleotides were affected by the original information directly and, therefore, a higher weight used; and the third nucleotide wobble feature was determined by certain natural choice mechanisms in evolutionary processes, indicating that this should be given a lower weight.

**Figure 5:**
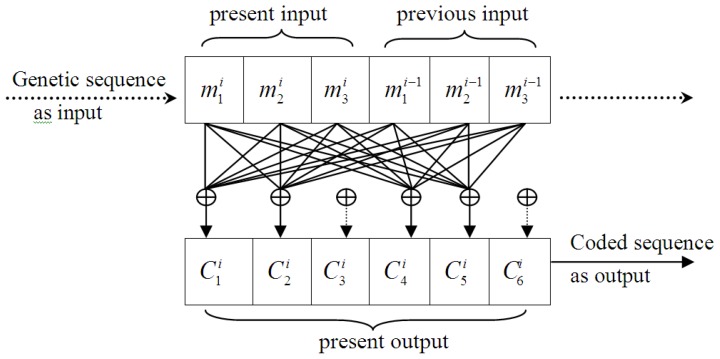
Designed (6,3,2) convolutional encoder.

The designed encoder is shown in Figure 5, where $C_{j}^{i}$ is the *j-th* bit of the output at time *i*. The output bits with a dotted arrow mean that they are determined by certain natural choice mechanism, according to the present hypothesis. The binary generator matrix was defined as:

(1) $$g^{1}=g^{2}=\left[\begin{array}{cccccc} 1 & 1 & 0 & 1 & 1 & 0\\ 1 & 1 & 0 & 1 & 1 & 0\\ 1 & 1 & 0 & 1 & 1 & 0 \end{array}\right]$$

where $g_{k,n}^{l}$ denoted whether the input data of the *k-th* row (*k* = 1,2,3) and the *l-th* column (*l* = 1,…,6) acted on the output $C_{n}^{i}$ (*n* = 1,…,6) at time *i*. When $g_{k,n}^{l}=1$, an influence existed (a solid line exists between $m_{k}^{i}$ and operator ⊕), and when 0, no direct influence was present (no solid line).

The output of encoder was:

(2) $$C=[m^{2}g^{1}+m^{1}g^{2}]$$

where *m*^2^ is the present 3 input symbols and *m*^1^ the previous 3 input symbols. The operation rules are listed in Table 3 [7].

### 3.2. Analysis Method

Considering model organisms, 12 prokaryote and 9 eukaryote DNA sequences were chosen, which possessed different GC contents, the latter chosen with the help of a taxonomic outline of the prokaryotes, Bergey’s Manual of Systematic Bacteriology [33] (Tables 1 and 2, respectively). GeneMark was used to analyze these sequences, downloaded from the NCBI, and coding strands picked out for further analysis. The first nucleotide of the start codon of an open reading frame (ORF) is defined as site 0, and before and after the initiation site, *M* nucleotides were taken out to obtain a sequence with a length of 2*M*.

The sequences were analyzed using the following steps:

Step 1 Digitizing the nucleotide sequences. The four nucleotides, *A*, *G*, *C* and *T*, were expressed as digital numbers, 0, 1, 2 and 3, respectively [7].
Step 2 Calculating the output of the convolutional code, using formula (2).
Step 3 Calculating the code distance. Code distance or Hamming distance, between two strings with equal length, is the number of positions for which the corresponding symbols are different. The first 3 numbers of the present convolutional output were compared with previous input data, *m*^1^, to calculate the code distance. The first code distance is remarked by *d*_1_1__.

One nucleotide was right-shifted [34] on the nucleotide sequence, the second subsequence with 6 nucleotides was taken out as the input to the encoder, and then, the operation is repeated. The code distance sequences of this nucleotide sequence can be described as:

(3) $$\begin{array}{ccccc} d_{1}: & d_{1_{1}} & d_{1_{2}} & \ldots & d_{1_{2M-5}} \end{array}$$

For example, TTTAAG (333001) was first picked out from TTTAAGCAA; its convolutional code output is 220220. The previous input data, 333, was compared with the first 3 numbers of its corresponding convolutional output (220), and the code distance was 3. Next, TTAAGC is picked out one nucleotide to the right and TAAGCA follows and so on.

Step 4 Calculating all ORFs of a DNA sequence. The same operation was performed for every ORF of an analyzed DNA sequence, and all code distance sequences of this DNA sequence were:
  (4) $$\begin{array}{ccccc} d_{1}: & d_{1_{1}} & d_{1_{2}} & \ldots & d_{1_{2M-5}}\\ \vdots & & & \vdots & \\ d_{r}: & d_{r_{1}} & d_{r_{2}} & \ldots & d_{r_{2M-5}} \end{array}$$
  where *r* is the total number of coding strands of the analyzed DNA sequence. Last, the average value of each site was calculated, described as:
  (5) $$\begin{array}{ccccccc} d_{average}: & d_{1\;average} & d_{2\;average} & \cdots & d_{k\;average} & \cdots & d_{(2M-5)\;average} \end{array}$$
  where *d_k average_* = (*d*_1_*k*__ + *d*_2_*k*__ +··· + *d_r_k__*)/*r*.
Step 5 Calculating the characteristic average code distance (CACD) of a DNA sequence. The new parameter, CACD, is defined to quantify the feature of code distance of a DNA sequence:
  (6) $$d_{characteristic}=(d_{1average}+d_{2average}+\cdots +d_{(2M-5)average})/(2M-5)$$

In the same way, nucleotide sequences near a DNA sequence termination site were analyzed, where site 0 was associated with the first nucleotide of a stop codon.

## 4. Conclusions

In this article, error-correction coding theory and consideration of codon degeneracy were combined to design a species-independent generator matrix of a convolutional code. A codon was treated as an informational unit, and a (6,3,2) convolutional code was designed, with considerations regarding codon context and the short-range dominance of bases correlation. Such a species-independent model may be more suitable for the simultaneous analysis of multiple sequences.

Twelve prokaryotic and nine eukaryotic DNA sequences were analyzed, and the translation initiation and termination sites and the SD sequence were identified and located. The effectiveness of the proposed model provided new proof of the value of codon context. In addition, the results also illustrated the relationship between a biological feature (GC content) and a code parameter (CACD), *i.e.*, the CACD of the analyzed species was approximately proportional to its GC content, particularly in prokaryotes, and is a feature that has not been previously reported. GC content is used as a basic feature for microorganism taxonomy, with similar GC content indicating greater relatedness, but it does not exhibit the permutation of bases in a DNA sequence, which contained more sophisticated genetic information. Therefore, other methods, such as sequence alignment, are needed to further explore taxonomic relationships [35]. The present results from the application of this model highlight its utility as an added taxonomic characteristic for use in studying the relationships of living organisms. Moreover, this new method provided the ability to reveal period-3 features in coding regions.

In addition, the proposed model has been used for the analysis of the similarities/dissimilarities of DNA sequences [36]. The simulations suggest that a convolutional code model could be a promising model for further bioinformatics analysis and encourage continued study of biological systems in terms of communication engineering theory.

It has also been noted that more research is required to address important remaining issues. (i) Attention should be paid to certain outside influences, such as horizontal gene transfer, which is a genetic exchange between different organisms or different organelles and which occurs frequently in prokaryotes and has recently also been identified in eukaryotes [37]. (ii) It is important to extend understanding of the differences among the CACDs of DNA sequences with similar GC content and, thus, improve the efficiency of this method. Investigations into these issues are underway.

## Figures and Tables

**Table 1:** Selected prokaryotes and their features.

NCBI Ref. Seq. Access Number	Selected Prokaryotes a	GC Content (%)	CACD b	CACD c
NC_006349	*Burkholderia mallei* ATCC 23344 chromosome 2	68	2.3504	1.9076
NC_009434	*Pseudomonas stutzeri* A1501	63	2.3062	1.9455
NC_003197	*Salmonella typhimurium* LT2	52	2.2336	1.8721
NC_000913	*Escherichia coli* str. K-12 substr. MG1655	50	2.2330	1.8712
NC_004088	*Yersinia pestis* KIM	47	2.2195	1.8624
NC_003098	*Streptococcus pneumoniae* R6	39	2.1547	1.7943
NC_004070	*Streptococcus pyogenes* MGAS315	38	2.1529	1.8028
NC_004350	*Streptococcus mutans* UA159	36	2.1524	1.7987
NC_002662	*Lactococcus lactis* subsp. lactis Il1403	35	2.1184	1.7827
NC_004461	*Staphylococcus epidermidis* ATCC 12228	32	2.1149	1.7699
NC_002758	*Staphylococcus aureus* subsp. aureus Mu50	32	2.1139	1.7658
NC_010163	*Acholeplasma laidlawii* PG-8A		2.1135	1.7632
		31	max − min = 0.2369	max − min = 0.1823

a All sequences are complete genome;

b CACD of the (6,3,2) convolutional code near initiation site;

c CACD of May *et al.*’s (5, 2) block code near initiation site.

CACD, characteristic average code distances.

**Table 2:** Selected eukaryotes and their features.

NCBI Ref. Seq. Access Number	Selected Eukaryotes a	GC Content (%)	CACD b	CACD c
NC_006070	*Yarrowia lipolytica* CLIB122 chromosome D	49	2.2267	1.9182
NW_045720	*Anopheles gambiae* str. PEST chromosome X	45	2.2350	1.8954
NC_008403	*Oryza sativa* (japonica cultivar-group) genomic DNA, chromosome 10	44	2.2777	1.8945
NT_011512	*Homo sapiens* chromosome 21, reference assembly	39	2.1900	1.8594
NC_001147	*Saccharomyces cerevisiae* chromosome XV	38	2.1537	1.8303
NC_001148	*Saccharomyces cerevisiae* chromosome XVI	38	2.1504	1.8347
NC_003075	*Arabidopsis thaliana* chromosome 4	36	2.2015	1.8409
NC_004353	*Drosophila melanogaster* chromosome 4	36	2.1966	1.8501
NC_003421	*Schizosaccharomyces pombe* 972h chromosome III	36	2.1814	1.8328
			max − min = 0.1273	max − min = 0.0879

a These sequences are complete sequences, with the exception of NW_045720, commented as whole genome shotgun sequence, and NT_011512, commented as reference assembly complete sequence;

b CACD of the (6,3,2) convolutional code near initiation site;

c CACD of May *et al.*’s (5, 2) block code near initiation site.

**Table 3:** Operation for addition and multiplication.

Addition Multiplication										
+	0	1	2	3		×	0	1	2	3
0	0	1	2	3		0	0	0	0	0
1	1	0	3	2		1	0	1	2	3
2	2	3	0	1		2	0	2	3	1
3	3	2	1	0		3	0	3	1	2
